# Enhanced recovery after surgery nursing program, a protective factor for stoma-related complications in patients with low rectal cancer

**DOI:** 10.1186/s12893-020-00978-3

**Published:** 2020-12-04

**Authors:** Weiling Shao, Honggang Wang, Qun Chen, Wen Zhao, Yulian Gu, Guoqin Feng

**Affiliations:** 1 Department of General Surgery, Taizhou People’s Hospital, Taizhou, China; 2 Department of Nursing, Taizhou People’s Hospital, No. 366 Taihu Road, Taizhou, 225300 Jiangsu Province China

**Keywords:** Low rectal cancer, Stoma-related complications, Enhanced recovery after surgery nursing program, Prognosis, Life of quality

## Abstract

**Background:**

This study aimed to investigate the association between enhanced recovery after surgery (ERAS) nursing program and stoma-related complications (SRCs) and prognosis in patients with low rectal cancer (LRC) undergoing abdominoperineal resection with sigmoidostomy.

**Methods:**

LRC patients who underwent elective abdominoperineal resection with sigmoidostomy between May 2016 and May 2019 were retrospectively enrolled. The occurrence of early major or minor SRCs (within postoperative 30 days) was set as the primary end-point. Clinicopathological variables and laboratory tests were compared between patients with or without SRCs. The univariate and multivariate logistic regression analyses were performed to investigate risk factors for SRCs. Hospitalization satisfaction-related and prognosis-related variables were compared between LRC patients with or without ERAS nursing program.

**Results:**

A total of 288 patients were enrolled and the incidence of SRCs was 26.7% (77/288). ERAS nursing program was the only independent risk factor for SRCs in LRC patients (OR 2.04, 95%CI 1.31–3.12, P = 0.016) by the multivariate logistic regression analysis. Moreover, ERAS nursing program was associated with higher hospitalization satisfaction rate, faster bowel function recovery, better psychological status, and higher quality of life.

**Conclusions:**

ERAS nursing program was a protective factor for SRCs and associated with improved prognosis in LRC patients undergoing elective abdominoperineal resection with sigmoidostomy.

## Background

Colorectal cancer (CRC) is the third most common cancer and the second leading cause of cancer-related death worldwide with over 1.8 million new cases and 881,000 cancer-related death in 2018 [1]. The incidence of low rectal cancer (LRC) among Chinese is reported to be 60–75% higher than Western populations [2, 3]. As for those LRC patients, abdominoperineal resection with sigmoidostomy is widely accepted as an effective and safe surgical strategy and considered as the standard treatment to decrease the risk of positive distal edge [4]. A stoma is a physical and psychological burden for the patients to accept this anatomical change. The reported rates of stoma-related complications (SRCs) are quite high, ranging from 21 to 70% [5, 6]. Furthermore, its real frequency is probably underestimated due to the autonomous management by stoma therapist without records. SRCs may have a great impact on the postoperative recovery, morbidity, mortality, and quality of life (QOL) [7–9]. Therefore, to investigate potential predictors and effective preventive measures for SRCs is of great clinical importance for prognosis improvement.

Enhanced recovery after surgery (ERAS), which was first put forward by Kehlet in the mid-1990s [10], has shown notably promising results in reducing the length of stay (LOS) and morbidity after colorectal surgery [11, 12]. A recent meta-analysis by Lau et al. has demonstrated that ERAS is associated with decreased LOS, postoperative complications, economic cost, and earlier gastrointestinal function recovery in colorectal surgery [13]. Nursing is a critically important part of ERAS and it is becoming more popular in clinical application. However, the predictive role of ERAS nursing program for SRCs remains unclear, which was the main goal of this present study.

## Methods

### Patients

This was a single-center retrospective study and it was approved by the Medical Institutional Ethics Committee of our hospital. We retrospectively recruited LRC patients who underwent elective abdominoperineal resection with sigmoidostomy at the Department of General Surgery, Taizhou Peoples’ Hospital between May 2016 and May 2019. Each patient was required to offer the signed informed consent. Inclusion criteria: (a) aged between 18 and 75 years; (b) with a histological diagnosis with LRC (within 8 cm from the anal verge); (c) abdominoperineal resection with sigmoidostomy. Exclusion criteria: (a) with surgical contraindication, e.g. distant metastasis, and severe obstruction; (b) undergoing emergency operations; (c) with malignancies in other systems; (d) with severe hematological, hepatic, kidney disorder, or autoimmune diseases; (e) with preoperative chemotherapy, radiotherapy, or targeted therapy for cancers; (f) with incomplete data. All the patients were under the perioperative management of the same surgical, anesthesia, and nursing team. Protocols of ERAS nursing program were according to the latest Chinese guidelines of ERAS (version 2016 and 2018). In brief, preoperative ERAS program education, personalized nutritional evaluation and support (based on NRS 2002 score), psychological counseling, psychological status evaluation, and improvement (reduce anxiety), no overnight fasting (clear fluids up to 2 h and solid food up to 6 h before the induction of anaesthesia) and carbohydrate loading drinks before the surgery (10% glucose solution oral administered 1000 ml 10 h and 400 ml 2 h before the induction of anaesthesia), preoperative bowel preparation (polyethylene glycol electrolyte powder the night before the surgery), venous thromboembolism prevention (based on Caprini score), intraoperative warming (maintain core body temperature ≥ 36 °C), avoidance of routine abdominal drains and nasogastric tubes, encouraged early postoperative mobilization (postoperative day 1), early catheter remove (postoperative day 1), dietary (fluids and enteral nutrition from postoperative day 1), pain (multimodal analgesis, combined with NSAIDs, reduce the use of opioid analgesics), sleep, and psychology management were recommended. Enrolled patients were followed up for at least three months from the surgical day.

The following data were extracted from our database and recorded: (1) demographic variables including age, gender, body mass index (BMI), smoking and drinking habits; (2) clinical baseline variables including serum carcinoembryonic antigen (CEA), American Society of Anesthesiologists (ASA) grade, Charlson Comorbidity Index [14], nutritional risk score (NRS)-2002 [15]; (3) pathological data including T stage, N stage, TNM stage, and pathologic differentiation; (4) treatment-related variables including surgical approach, ERAS nursing program, preoperative stoma localization, operation time, estimated blood loss, height of stoma, and base area of stoma; (5) laboratory tests including hemoglobin (Hb), albumin (Alb), white blood cell (WBC), hematocrit (Hct), C-reactive protein (CRP), creatinine, and urea; (6) Chinese hospitalization satisfaction-related variables (11 items) including safety, environment, accessibility, respect, nursing technique, comfortableness, health education, communication, emotion support, participation in nursing, and discharge and referral; (7) prognosis-related variables including time to first exhaust and defecation, LOS, self-rating anxiety scale (SAS) scores, self-rating depression scale (SDS) scores [16], Gastrointestinal Quality of Life Index (GIQLI) [17] including physiological function, mental health, social function, and subjective symptoms at 3 months after the surgery.

As described by previous reports, the primary observational end-point was the occurrence of early major or minor SRCs (within postoperative 30 days). In brief, major SRCs included stoma prolapse, parastomal hernia, stricture, fistula, retraction, ischemia, and bleeding. Minor SRCs included skin alterations according to the classification by SACS™ instrument (ConvaTec, Reading, Berkshire, UK) for assessing peristomal skin lesion [18].

The base area of stoma was measured postoperatively by nurses calculating from the horizontal and vertical size of the stoma base. The pathological TNM stage was identified according to the criteria by the American Joint Committee on Cancer/International Union Against Cancer (7th edition).

### Statistical analysis

GraphPad Prism 8.0 (GraphPad Inc., CA, USA) and SPSS 19.0 (SPSS Inc., Chicago, IL, USA) were used for data analysis. Data are expressed as number (n) with percentage (%) or mean with standard deviation (SD) as appropriate. Data are analyzed using the Chi-square test, Student’s *t*-test, and Mann–Whitney U-test as appropriate. The univariate and multivariate logistic regression analyses were performed to investigate risk factors for SRCs. A two-sided P value < 0.05 was considered statistically different.

## Results

### Patient characteristics

Three hundred and thirty-three LRC patients who underwent elective abdominoperineal resection with sigmoidostomy were initially enrolled. Forty-five were excluded due to the following reasons (10 with surgical contraindication, 6 undergoing emergency operations, 5 with malignancies in other systems, 9 with severe hematological, hepatic, or kidney disorder, and 15 with incomplete data) and two hundred and eighty-eight subjects were included in the analysis. The mean age of the cohort was 66.2 years and the majority (69.4%, 200/288) were male patients. The total incidence of SRCs was 26.7% (77/288). The clinicopathological characteristics associated with SRCs are shown in Table 1. There were no significant differences in gender, ASA grade, smoking and drinking habits, serum CEA, T stage, N stage, TNM stage, pathologic differentiation, surgical approach, estimated blood loss, and height of stoma between patients with and without SRCs (P > 0.05). Patients in the SRCs group seemed to have a higher percentage of elderly (≥ 65 years) (P = 0.048) and overweight (BMI > 24.5) (P = 0.031). Patients with a higher NRS 2002 score (≥ 3) (P = 0.015), the base area of stoma (P = 0.019), and longer operation time (P = 0.028) were associated with SRCs occurrence. Moreover, significant differences were noted for ERAS nursing program (P = 0.010) and preoperative stoma localization (P = 0.040) between patients who developed SRCs or not.

**Table 1:** Clinicopathological variables associated with SRCs in LRC patients

Variables	SRCs		*P*-value
	Yes	No	
Number, n (%)	77 (26.7)	211 (73.3)	–
Age (year), n (%)	–	–	0.048*
≥ 65	47 (61.0)	101 (47.9)	–
< 65	30 (39.0)	110 (52.1)	–
Gender, n (%)	–	–	0.114
Male	48 (62.3)	152 (72.0)	–
Female	29 (37.7)	59 (28.0)	–
BMI (kg/m^2^), n (%)	–	–	0.031*
≥ 24.5	31 (40.3)	57 (27.0)	–
< 24.5	46 (59.7)	154 (73.0)	–
ASA grade, n (%)	–	–	0.668
I/II	49 (63.6)	140 (66.4)	–
III/IV	28 (36.4)	71 (33.6)	–
Charlson Comorbidity Index	3.2 ± 0.8	2.5 ± 0.6	< 0.001
NRS 2002 score, n (%)	–	–	0.015*
≥ 3	17 (22.1)	23 (10.9)	
< 3	60 (77.9)	188 (89.1)	
Active smoker, n (%)	1 0(13.0)	26 (12.3)	0.880
Heavy drinker, n (%)	8 (10.4)	22 (10.4)	0.993
Serum CEA (ng/mL), n (%)	–	–	0.686
≥ 5	36 (46.8)	93 (44.1)	–
< 5	41 (53.2)	118 (55.9)	–
T stage, n (%)	–	–	0.409
T1/2	23 (29.9)	74 (35.1)	–
T3/4	54 (70.1)	13 7(64.9)	–
N stage, n (%)	–	–	0.163
Negative	45 (58.4)	142 (67.3)	–
Positive	32 (41.6)	69 (32.7)	–
TNM stage, n (%)	–	–	0.663
I/II	46 (59.7)	120 (56.9)	–
III	31 (40.3)	91 (43.1)	–
Pathologic differentiation, n (%)	–	–	0.936
Well/moderate	62 (80.5)	169 (80.1)	–
Poor/mucinous	15 (19.5)	42 (19.9)	–
Surgical approach, n (%)	–	–	0.371
Laparoscopic	54 (70.1)	159 (75.4)	–
Laparotomy	23 (29.9)	52 (24.6)	–
ERAS nursing program, n (%)	–	–	0.010*
Yes	41 (53.2)	147 (69.7)	–
No	36 (46.8)	64 (30.3)	–
Preoperative stoma localization, n (%)	10 (13.0)	51 (24.2)	0.040*
Operation time (min)	167.3 ± 37.4	155.9 ± 39.3	0.028*
Estimated blood loss (ml)	138.1 ± 50.5	146.5 ± 55.7	0.247
Height of stoma (mm)	10.7 ± 1.3	11.0 ± 1.2	0.067
Base area of stoma (cm^2^)	9.0 ± 1.1	8.7 ± 0.9	0.019*

*ERAS* enhanced recovery after surgery, *LRC* low rectal cancer, *SRCs* stoma-related complications, *BMI* body mass index, *ASA* American Society of Anesthesiologists, *NRS* nutritional risk score, *CEA* carcinoembryonic antigen

*P value < 0.05

### Laboratory variables associated with SRCs

Table 2 presents the preoperative levels of laboratory variables in patients with or without SRCs. The results indicated that patients with SRCs had higher rates of abnormal Alb (< 35.0 g/L) and CRP (> 0.8 mg/L) expressions (P = 0.025 and 0.038, respectively). No statistically significant differences were observed concerning Hb, WBC, creatinine, and urea between these two groups (P > 0.05).

**Table 2:** Laboratory tests associated with SRCs in LRC patients

Variables	SRCs		*P*-value
	Yes	No	
Number, n (%)	77 (26.7)	211 (73.3)	*–*
Hb (g/L)	109.7 ± 7.7	110.3 ± 8.1	0.722
WBC (× 10^9^/L)	7.5 ± 2.0	7.1 ± 1.8	0.107
Albumin (g/L), n (%)	–	–	0.025*
< 35.0	21 (27.3)	33 (15.6)	
≥ 35.0	56 (72.7)	178 (84.4)	
CRP (mg/L), n (%)	–	–	0.038*
> 0.8	33 (42.9)	63 (29.9)	–
≤ 0.8	44 (57.1)	148 (70.1)	–
Creatinine(umol/L)	71.8 ± 9.8	72.5 ± 10.1	0.600
Urea(mmol/L)	6.0 ± 1.3	5.9 ± 1.2	0.541

*LRC* low rectal cancer, *SRCs* stoma-related complications, *Hb* hemoglobin, *Alb* albumin, *WBC* white blood cell, *Hct* hematocrit, *CRP* C-reactive protein

*P value < 0.05

### Risk factors for SRCs

To identify potential risk factors for SRCs, the univariate and multivariate logistic regression analyses were performed. As illustrated in Table 3, five variables (age, NRS 2002 score, ERAS nursing program, Alb, and CRP) were risk factors associated with SRCs (P < 0.05). Our results from the multivariate logistic regression analysis indicated ERAS nursing program as the only independent risk factor for SRCs in LRC patients (OR 2.04, 95%CI 1.31–3.12, P = 0.016).

**Table 3:** Risk factors associated with SRCs in LRC patients by univariate and multivariate logistic regression analyses

Variables	Univariate multivariate			
	OR (95%CI)	*P* value	OR (95%CI)	*P* value
Age (≥ 65 vs < 65)	1.75 (1.09–2.84)	0.014*	1.27 (0.82–1.98)	0.241
BMI (≥ 24.5 vs < 24.5)	2.31 (0.72–7.23)	0.169		
Charlson Comorbidity Index (III vs II)	2.10 (0.75–5.51)	0.121		
NRS 2002 score (≥ 3 vs < 3)	1.46 (1.02–2.05)	0.028*	1.03 (0.95–1.12)	0.304
ERAS nursing program (no vs yes)	2.47 (1.42–4.01)	0.009*	2.04 (1.31–3.12)	0.016*
Preoperative stoma localization (no vs yes)	1.11 (0.81–1.57)	0.489		
Operation time (≥ 162 vs < 162)	1.50 (0.33–6.41)	0.573		
Base area of stoma (≥ 8.8 vs < 8.8)	1.68 (0.37–7.71)	0.467		
Albumin (< 35.0 vs ≥ 35.0)	1.78 (1.04–2.89)	0.012*	2.05 (0.65–6.49)	0.221
CRP (> 0.8 vs ≤ 0.8)	1.54 (1.01–2.35)	0.037*	1.26 (0.93–1.71)	0.122

*ERAS* enhanced recovery after surgery, *LRC* low rectal cancer, *SRCs* stoma-related complications, *BMI* body mass index, *NRS* nutritional risk score, *CEA* carcinoembryonic antigen, *CRP* C-reactive protein, *OR* odds ratio, *CI* confidence interval

*P value < 0.05

### Hospitalization satisfaction-related variables and ERAS nursing program

Based on the presence of ERAS nursing program, enrolled patients were categorized into two groups (188 with ERAS nursing program and 100 without). The eleven hospitalization satisfaction-related variables were compared between patients with or without ERAS nursing program. As shown in Table 4, patients who underwent ERAS nursing program had higher scores of 4 items (comfortableness, communication, emotional support, and participation in nursing) than those without ERAS nursing program (P < 0.05).

**Table 4:** ERAS nursing program and hospitalization satisfaction-related variables in LRC patients

Hospitalization satisfaction-related	ERAS nursing program		*P*-value
variables	Yes	No	
Number, n (%)	188 (65.3)	100 (34.7)	–
Safety	14.1 ± 2.3	13.9 ± 2.5	0.496
Environment	8.9 ± 0.7	8.8 ± 1.0	0.323
Accessibility	19.5 ± 0.7	19.4 ± 0.8	0.273
Respect	14.8 ± 1.6	14.5 ± 1.9	0.157
Nursing technique	4.9 ± 0.6	4.8 ± 0.7	0.205
Comfortableness	24.6 ± 0.9	24.2 ± 1.2	0.002*
Health education	9.6 ± 0.6	9.5 ± 0.8	0.313
Communication	14.4 ± 1.1	13.4 ± 1.3	< 0.001*
Emotional support	4.8 ± 0.4	4.3 ± 0.6	< 0.001*
Participation in nursing	14.7 ± 0.9	14.3 ± 1.1	0.001*
Discharge and referral	14.3 ± 0.8	14.2 ± 1.0	0.356

*ERAS* enhanced recovery after surgery, *LRC* low rectal cancer

*P value < 0.05

### Prognosis-related variables and ERAS nursing program

The prognosis-related variables between ERAS nursing and non-ERAS nursing program groups are listed in Table 5. The time to first exhaust (P = 0.036) and defecation (P = 0.002), and LOS (P = 0.007) of patients in the ERAS nursing program group were all significantly lower than those in the non-ERAS nursing program group. Moreover, patients who underwent ERAS nursing program seemed to have better psychological statuses (higher SAS and SDS scores) and higher quality of life (higher GIQLI scores) than those who did not undergo ERAS nursing program (P < 0.05).

**Table 5:** ERAS nursing program and prognosis-related variables in LRC patients

Prognosis-related	ERAS nursing program		*P*-value
variables	Yes	No	
Number, n (%)	188 (65.3)	100 (34.7)	–
Time to first exhaust (day)	2.1 ± 0.8	2.3 ± 0.7	0.036*
Time to defecation (day)	3.3 ± 0.9	3.7 ± 1.2	0.002*
LOS (day)	6.9 ± 1.3	7.4 ± 1.8	0.007*
SAS scores	53.1 ± 6.7	51.4 ± 5.0	0.027*
SDS scores	52.3 ± 5.5	50.7 ± 4.4	0.005*
GIQLI scores	–	–	–
Subjective symptoms	65.4 ± 5.7	63.5 ± 6.1	0.009
Physiological function	19.1 ± 2.3	18.3 ± 1.7	0.002
Mental health	13.5 ± 1.9	12.9 ± 1.6	0.008
Social function	13.1 ± 2.1	12.5 ± 1.8	0.016

*ERAS* enhanced recovery after surgery, *LRC* low rectal cancer, *LOS* length of stay, *SAS* self-rating anxiety scale, *SDS* self-rating depression scale, *GIQLI* Gastrointestinal Quality of Life Index

*P value < 0.05

## Discussion

To our knowledge, this was the first study to indicate ERAS nursing program as an independent risk factor for SRCs. The operation of stoma formation is commonly performed for patients with malignancy and inflammatory bowel disease (IBD) [19]. Stoma formation is usually performed after a long and complex surgical operation. This procedure is simply undertaken, but it is associated with significant morbidity, complex and life-threatening consequences [20]. The incidence of SRCs in LRC patients after abdominoperineal resection with sigmoidostomy was demonstrated to be 26.7% in our cohort. A previous single-center prospective study by Pearson et al. have reported an overall SRCs rate of 23.5% among 408 patients following ostomy surgery over two years [20]. Moreover, the incidences between the elective and emergency operation groups were quite similar [20]. Another study by Arumugam et al. has elaborated an incidence of 50.5% for one or more SRCs in a prospective study of 97 patients [21]. We considered that the differences in stoma location (colostomy or ileostomy), patient characteristics, surgical types, stoma nursing, and SRCs definitions were probably the main explanations for different incidences in different reports.

Various variables have been identified as independent risk factors for SRCs, including ASA grade [22], age, ileostomy, and loop stomas [23], male sex, and ileostomy creation [24]. Our results firstly demonstrated that ERAS nursing program was associated with decreased SRCs rates. A previous study by Forsmo et al. revealed that SRCs were not significantly different between ERAS and non-ERAS groups although with a tendency [25], which was not quite in accordance with our results. The stoma nursing has great impacts on the occurrence of SRCs, especially stoma infection and fecal dermatitis.

Based on our results, it appears that ERAS nursing program provided significant benefit to LRC patients undergoing abdominoperineal resection with sigmoidostomy. Our results demonstrated that the ERAS nursing program can be successfully implemented in a single-center municipal hospital. With the adoption of ERAS nursing program, patients were more likely to have better hospitalization satisfaction for the items of comfortableness, communication, emotional support, and participation in nursing. Furthermore, patients who underwent ERAS nursing program had an improved prognosis, which was manifested by earlier gastrointestinal function recovery (shorter time to first exhaust and defecation), shorter LOS, higher SDS and SAS scores, higher QOL (higher GIQLI scores). The decreased narcotic use in the ERAS program partially explains enhanced bowel functional recovery due to the well-known effects of narcotics [26]. Preoperative nutritional evaluation and support by ERAS nursing program can reduce the incidence of insulin resistance and postoperative hyperglycemia, and reduce preoperative anxiety. The improvement of nutritional status is widely reported to be associated with improved systemic immune function and better prognosis [27, 28]. Stoma formation is also a great threat to QOL for LRC patients due to the low acceptance rate, troublesome stoma nursing, and psychological burden. To some extent, the decreased SRCs rate correlates with increased QOL. The importance of guarantee a further rectal continence and function in patients who underwent ostomy is mandatory. But several others factors that could alter physiologic defecation are implied in fecal continence. Bocchini et al. [29] suggest the criteria for patients selection, evaluation of clinical presentation of defecatory disorders, appropriate education about concepts of pelvic anatomy and defecation physiology, and protocols of pelvic floor rehabilitation for defecation disorders, including fecal incontinence. Based on their reports, Brusciano et al. [30] propose a modified physiatric assessment (combination of chest, abdomen, vertebral column, and perineum) instead of solely on the function and integrity of the pelvic floor. Interestingly, Gambardella et al. [31] hold the same view that the muscular synergies evaluation (a clinical-physiatric evaluation) can serve as a predictive parameter to identify incontinent patients amenable for rehabilitation treatment. In summary, a systemic evaluation is recommended before pelvic floor rehabilitation for defecation disorders.

There are some limitations to this study. First, this is a single-center study with a relatively small sample size. Second, the retrospective nature and long-time inclusion period generate some uncontrollable biases. Third, the involved mechanisms remain unknown. Last, only LRC patients were enrolled and whether our conclusions apply to other stoma operations remain uncertain. Considering ERAS nursing program can reduce postoperative SRCs, improve the hospitalization satisfaction and QOL, it would be generally desirable.

## Conclusion

In conclusion, ERAS nursing program was a protective factor for SRCs and associated with improved prognosis in LRC patients undergoing elective abdominoperineal resection with sigmoidostomy.

## Data Availability

Please contact the corresponding author (Guoqin Feng, email: fengguoqin.tz@outlook.com) for data requests.

## Abbreviations

ERAS: Enhanced recovery after surgery
SRCs: Stoma-related complications
LRC: Low rectal cancer
QOL: Life of quality
CRC: Colorectal cancer
LOS: Length of stay
BMI: Body mass index
CEA: Carcinoembryonic antigen
NRS: Nutritional risk score
Hb: Hemoglobin
Alb: Albumin
WBC: White blood cell
Hct: Hematocrit
CRP: C-reactive protein
SAS: Self-rating anxiety scale
SDS: Self-rating depression scale
GIQLI: Gastrointestinal Quality of Life Index
SD: Standard deviation
IBD: Inflammatory bowel disease
OR: Odds ratio
CI: Confidence interval
